# Editorial: Control and prevention of tropical diseases by advanced tools and the One Health approach

**DOI:** 10.3389/fmicb.2023.1289224

**Published:** 2023-09-25

**Authors:** Yang Hong, Kokouvi Kassegne, Moses Okpeku, Bin Zheng, Jun-Hu Chen

**Affiliations:** ^1^National Health Commission of the People's Republic of China (NHC) Key Laboratory of Parasite and Vector Biology, National Institute of Parasitic Diseases, Chinese Center for Diseases Control and Prevention (Chinese Center for Tropical Diseases Research), World Health Organization (WHO) Collaborating Center for Tropical Diseases, National Center for International Research on Tropical Diseases, Shanghai, China; ^2^Hainan Tropical Diseases Research Center (Hainan Sub-Center, Chinese Center for Tropical Diseases Research), Haikou, China; ^3^School of Global Health, Chinese Centre for Tropical Diseases Research, Shanghai Jiao Tong University School of Medicine, Shanghai, China; ^4^Discipline of Genetics, School of Life Sciences, University of KwaZulu-Natal, Durban, South Africa; ^5^School of Basic Medicine and Forensics, Hangzhou Medical College, Hangzhou, China

**Keywords:** tropical diseases, advanced tools, One Health, diagnosis, vaccine, drug

One Health is described as the collaborative efforts of multiple disciplines working locally, nationally, and globally to attain optimal health for humans, animals and the environment. It recognizes the health of humans, domestic and wild animals, plants, and the wider environment (including ecosystems) which are closely linked and interdependent. The final aim of One Health is to sustainably balance and optimize the health of humans, animals and ecosystems. It is a collaborative global approach to understanding and managing risks for planetary health and encouraging a more sustainable ecosystem balance.

In recent years, many factors have changed interactions between humans, animals, plants, and the environment. These changes have led to the spread of existing or known (endemic) and new or emerging zoonotic diseases that can spread between animals and humans. Every year, millions of humans and animals are affected by zoonotic diseases worldwide. Emerging and re-emerging zoonotic diseases represent a public health challenge of global concern. They include a large group of tropical diseases (TDs), many of which are zoonotic in nature. TDs are especially common in tropical areas, including several countries in Africa, Asia, and Latin America, where humans do not have full access to clean water or safe ways to dispose of human waste. TDs include several parasitic, viral, and bacterial diseases that are transmitted directly from person to person or through vectors (e.g., insects and mollusks) and cause more than one million human deaths globally. Affecting the world's poorest humans, TDs impair physical and cognitive development, contribute to mother, child illness, and death, affect humans' ability to work, and limit productivity at work. As a result, TDs trap the poor in a cycle of poverty and disease.

There are five parasite species that regularly cause malaria in humans, and two of these species – *Plasmodium falciparum* and *P. vivax* – account for large morbidity and mortality, particularly in sub-Saharan Africa, where the greatest burden of the disease is borne. In 2020, nearly half of the world's population was at risk of malaria. Developing malaria vaccines is a good choice to prevent the occurrence and spread of this disease. Sun et al. used the reverse genetics system to construct recombinant vesicular stomatitis virus (rVSVs), which is a single-stranded negative-strand RNA virus widely used as a vector for virus or cancer vaccines, expressing apical membrane protein 1 (AMA1), rhoptry neck protein 2 (RON2), and reticulocyte-binding protein homolog 5 (RH5). These proteins are required for *P. falciparum* invasion. The authors found that a VSV-based viral vaccine approach for malaria exhibited efficacy in inducing specific humoral and cellular immune responses as well as inhibiting *P. falciparum* invasion. *Plasmodium*-specific IgG levels and lymphocyte proliferation could be significantly increased after vaccination. Prime-boost regimens with VSV-PyAMA1 and VSV-PyRON2sp could significantly improve the levels of IL-2 and IFN- γ produced by CD${}_{4}^{+}$ and CD${}_{8}^{+}$ T cells and suppress invasion *in vitro*. Meanwhile, the rVSV prime-protein boost regimen significantly increased *Plasmodium* antigen-specific IgG levels in the serum of mice. In the immunoprotection experiment, the mice immunized by rVSV prime protein boost displayed a better protective efficacy against *P. yoelii* 17XL compared to traditional antigen immunization.

Liu et al. examined genomic epidemiology during the pre-elimination stage by retrospectively reporting whole-genome sequence variation of 10 *P. vivax* isolates from inland China in the 2010s, which were highly structured compared to the surrounding area, with a single potential ancestor. Some genes, such as ugt, krs1, and crt, could be used as selection signatures in drug resistance. The proportion of susceptible isolates (wild-type dhps and dhfr-ts) fluctuated in response to the prohibition of sulfadoxine-pyrimethamine. The findings of the study also implied that superinfection or cotransmission events are rare in low-endemic circumstances.

The filarial nematodes that cause these debilitating diseases are transmitted by blood-feeding insects and produce chronic and long-term infection through suppression of host immunity. Lymphatic filariasis and onchocerciasis are parasitic helminth diseases that constitute a serious public health issue in tropical regions, and the two diseases are mainly treated via mass drug administration (MDA), such as ivermectin, albendazole and diethylcarbamazine.

OXF and flubendazole (FBZ) are two benzimidazoles that selectively target the β-tubulin subunits of nematodes, and they have shown macrofilaricidal activities against different filarial species. Risch et al. demonstrated the significant contributions of the immune system during anti-filarial treatment with OXF and FBZ using the *Litomosoides sigmodontis* rodent model. OXF and FBZ induced marked differences in both spleen and thoracic cavity cell compositions with distinct differential patterns in various immunodeficient mouse strains.

IL-5, but not IL-4 or IL-33, combined with OXF could improve the macrofilaricidal efficacy, and a shortened 3-day treatment could be boosted for worm burden reduction. The number of microfilaria-positive animals decreased, and no lung pathological changes were observed in treated mice. Thus, some components of the host immune system could support the filaricidal effect of benzimidazoles *in vivo* and present an opportunity to boost treatment efficacy. These findings may yield more treatment options for human filarial patients.

*Dirofilaria immitis*, known as heartworm or dog heartworm, is a small thread-like parasitic roundworm. It is a type of filarial worm that causes dirofilariasis, which is a major emergent veterinary parasitic infection and a human zoonosis. Marriott et al. demonstrated that multiple lymphopenic immunodeficient mouse strains with ablation of the interleukin-2/7 common gamma chain (γc) are susceptible to the initial tissue larval development phase of *D. immitis*. This model provides universal access to accurate and facile PK-PD assessments of preventative *D. immitis* drug candidate responses against the prophylactic L3–L4 larval target. Meanwhile, it could avoid the risk of welfare issues and allow for rapid assessments. In the future, it might reduce the requirements for long-term cat and dog experimentation in heartworm studies.

*Toxoplasma gondii* (*T. gondii*) is an obligate intracellular parasite that can infect virtually all warm-blooded animals, including humans, cats, and birds. It threatens one-third of the world's population because of its broad host range, high infection rate, and benign coexistence with the host. Although various vaccination strategies have been used to develop an effective toxoplasmosis vaccine, there is still no safe and effective vaccine for *T. gondii*. To date, only Toxovax^®^ has been approved in a few regions for reducing the losses in sheep farming caused by congenital toxoplasmosis. Li et al. used TGGT1_316290 (TG290) as a potential vaccine target to construct TG290 mRNA-LNPs by the lipid nanoparticle (LNP) technology. After vaccination, TG290 mRNA-LNPs elicit humoral and cellular immune responses, enhanced cytokine production, and evoked DCs and T lymphocytes. TG290-specific total IgG and subtype IgG1 and IgG2a antibodies and cytokines (IFN-γ, IL-12, IL-4, and IL-10) were significantly elevated. Cytokine-related transcription factors, such as T-Box 21 (T-bet), nuclear factor kappa B (NF-kB) p65, and interferon regulatory factor 8 (IRF8) subunit, were also overexpressed. Meanwhile, TG290 mRNA-LNP vaccination prolonged the survival time of *T. gondii*-infected BALB/c mice. TG290 might be a potential candidate for an anti-*T. gondii* vaccine.

Babesiosis is globally distributed, and numerous wild and domestic animals may serve as infection reservoir hosts. The disease is mainly caused by *Babesia microti*, which is a tick-transmitted protozoan hemoparasite, and it usually induces a serious public health concern. Song et al. found that nearly 40% of erythrocytes could change their structure and shrink in *B. microti*-infected BALB/c mice. The infection could also cause significant splenomegaly, severe anemia, a massive loss of late erythroblasts and induce eryptosis. However, the population of early erythroblasts was identified to increase in both the bone marrow and spleen, which played a critical protective role in controlling *B. microti* infection and preventing anemia.

Schistosomiasis caused by three main species of schistosomes (trematode blood flukes) infecting humans, *Schistosoma mansoni, S. haematobium*, and *S. japonicum*, affects more than 230 million people worldwide. Diagnosis and treatment are the two effective methods to control the disease.

Mu et al. set up a urine-based enzyme-linked immunosorbent assay (ELISA) using SjSAP4 + Sj23-LHD as detection antigens. The diagnostic performance of the urine ELISA was evaluated in a human cohort recruited from areas in the Philippines that are moderately endemic for schistosomiasis japonica. Compared with other diagnostic tests, the urine ELISA showed a 47.2–56.9% sensitivity and a 50.7–55.2% specificity in the detection of *S. japonicum* infection. Although urine-based methods are convenient and highly acceptable non-invasive methods for clinical sample collection, they show insufficient sensitivity compared with corresponding serum-based methods. This disadvantage will limit its use in subjects with low worm burdens in rural schistosome-endemic areas post mass drug administration in the Philippines.

To date, the most effective drug for the treatment of schistosomiasis is still praziquantel (PZQ) in the clinic without any backup drugs. The neurotransmitter glutamate is involved in many physiological functions. Glutamate neuronal signaling can interact with various cell surface receptors for signal transduction, including ionotropic gated channels and metabotropic glutamate receptors (mGluRs, also known as GRMs). Thus, they might be promising drug targets for schistosomiasis.

Wang et al. identified two putative glutamate-specific mGluRs (GRM7 and GRM) in *S. japonicum*. SjGRM is mainly located in the gonads of both males and females, and SjGRM7 is principally found in the nerves and gonads of males and gonads of females. The expression of SjGRM was relatively stable during schistosome development in the definitive host. SjGRM7 was first downregulated before 26 dpi and then upregulated after 26 dpi. After dsRNA interference of SjGRM7 *in vivo*, the development of worms and egg production were affected, and host liver granulomas and fibrosis were alleviated. RNA sequencing data implied that SjGRM7 propagates its signals through the G protein-coupled receptor signaling pathway to promote nervous system development. This study prompted further drug development research to treat *S. japonicum*, and SjGRM7 might be a potential anti-schistosomiasis target.

Skin-associated lymphoid tissue is crucial for parasite control because the skin of mammals accomplishes complex physiological and immunological functions. Epiddermal Langerhans cells (LCs), favor the generation of *Leishmania*-specific T cells *in vivo*, and LCs are involved in adaptive immunity against *Leishmania* parasites, which are spread by the bite of phlebotomine sand flies.

Nerb et al. thought that the immunological attributes of LCs under “microbiome-free” and “microbiome-containing” sand fly or syringe conditions were not systematically compared. The role of LCs in adaptive immunity and the generation of long-lasting immunological memory need to be deciphered in detail. Natural transmission of *Leishmania* parasites is also not sterile. Using novel vector-associated “natural” microbial adjuvants might be an improvement of alternative rationally designed vaccination strategies for *Leishmania* parasites in the future.

Many species of mosquitoes can ingest pathogens while biting and transmit them to future hosts. Mosquitoes are important vectors for the transmission of vector-borne diseases, such as malaria, dengue or Zika.

Musah et al. identified that many open-source data sources from Brazil could be harnessed as risk factors for the spatial prediction of mosquito occupancy or infestation. They could be brought together and reproducibly implemented using the MAXENT algorithm within a Brazilian context. To address the problems of data paucity and avoid potential biases that are typically found in studies using open source datasets, the use of novel bespoke technologies, such as smartphone applications, might be considered a better method for collecting primary entomological data. This will improve a study's internal and external validity. The thread and reproducible method are applicable to different mosquito species and other areas in the Global South with similar environmental and socioeconomic conditions.

In summary, this themed Research Topic advances our knowledge of controlling, preventing, or eliminating TDs under the One Health concept. Many great efforts in epidemiology, vector control, and the use of drugs have been very helpful in bringing reduced incidence alongside great advancements toward TD eradication. Moreover, environmental factors greatly influence the prevalence of TDs. More effective and environmentally friendly strategies are needed to support TDs control. These studies provide useful information to better control TDs and to achieve the final aim of One Health.

## Author contributions

YH: Conceptualization, Writing—original draft. KK: Writing—review and editing. MO: Writing—review and editing. BZ: Writing—review and editing. J-HC: Conceptualization, Funding acquisition, Writing—review and editing.

